# Characterization of aortic endothelial dysfunction in ovariectomized allergic asthmatic mice

**DOI:** 10.1371/journal.pone.0352768

**Published:** 2026-07-22

**Authors:** Shinichi Asano, Alexandra P. Crawford, Cameron C. Felty, Amanda S. Hatcher, Abigail R. Patterson, Marina Diioia, Dovenia S. Ponnoth

**Affiliations:** 1 Department of Biomedical Sciences, West Virginia School of Osteopathic Medicine, Lewisburg, West Virginia, United States of America; 2 Department of Clinical Science, West Virginia School of Osteopathic Medicine, Lewisburg, West Virginia, United States of America; 3 Department of Chemistry and Biochemistry, West Virginia Wesleyan College, Buckhannon, West Virginia, United States of America; Cinvestav-IPN, MEXICO

## Abstract

Clinical observations suggest that sex hormones may have a role in female asthma-related cardiovascular diseases (CVD), however, the mechanism by which sex hormones may influence CVD in asthmatics is unknown. The goal of this study was to determine if ovariectomized (OVX) mice with asthma alter asthma severity biomarkers and vascular reactivity. Female C57/BL6 mice were assigned to 4 groups: 1) sham vehicle control (VC), 2) OVX VC, 3) sham asthma, and 4) OVX asthma. Asthmatic groups were sensitized with 30 μg ovalbumin suspended in Imject alum by i.p. injections followed by 5% ovalbumin in saline for aerosol challenges. Vehicle groups received an identical treatment without ovalbumin. In the lungs, the eosinophil population was significantly higher in OVX asthma mice than in sham asthma mice (58 ± 9 vs. 76 ± 5%). Similarly, the OVX asthma group had significantly higher plasma anti-IgE (8.98 ± 1.66 vs. 27.5 ± 2.07 ng/mL) and elevated cytokines in bronchoalveolar lavage fluids. Isometric tension experiments demonstrated that maximal acetylcholine-induced aortic relaxation was significantly reduced by approximately 24% in both asthmatic mice groups compared with respective vehicle control mice. Similarly, the nitric oxide component of Ach-induced relaxation was significantly reduced in both asthma groups, but there were no differences between sham and OVX asthmatic mice. Our data demonstrated that OVX asthmatic mice developed exacerbated allergic lung responses, and lung-initiated inflammation can extend to impair endothelial function in the aorta.

## Introduction

Asthma is a complex airway inflammatory disorder that affects people of all ages, sexes, races, ethnicities, and socioeconomic backgrounds. In the United States, the Centers of Disease Control and Prevention state that approximately 25 million people were affected by asthma in 2021 [1]. Asthma is more likely to occur in adolescent males than females, however, this sex specific incidence of asthma reverses in adulthood. In adults, females have an increased prevalence of asthma, and importantly, they suffer from a more severe form of asthma compared to men [2]. This disorder is unique in the female population as perimenstrual asthma is characterized by a significant worsening of symptoms just before, or during menstruation when both estrogen and progesterone levels are low [3]. In addition, recent clinical evidence suggests that female-specific asthma symptoms are associated with increased cardiovascular disease (CVD) risk [4,5]. These clinical observations suggest that female sex hormones may have a role in female-specific asthma-related CVD.

The mechanisms in question are thought to relate to, at least in part, sex hormones that alter inflammatory responses to asthma. In several preclinical studies, female mice showed increased ovalbumin-sensitization induced type 2 immune responses such as eosinophilia, and increased IL-5, and IL-13 in lungs compared to male mice [6–8]. However, results from asthma studies utilizing a rodent ovariectomy (OVX) model have been contradictory. In one study, OVX was reported to have a protective role on lung allergic responses such as decreased eosinophilia, IL-4, IL-5 and IL-13 in bronchoalveolar lavage (BAL) fluid compared to ovary intact female asthmatic mice [9]. On the other hand, another study has shown completely the opposite results, demonstrating OVX augmented airway inflammation and lung allergic responses [10]. This study showed that OVX resulted in exacerbated lung inflammation that was rescued by estrogen replacement, but not progesterone supplementation. Finally, another study showed that the effect of OVX on classic Th2 immune responses varies depending on the timing of OVX surgeries relative to antigen sensitization [11]. Thus, although there is a consensus that OVX asthma models alter lung inflammation, our understanding of the role of sex hormones in asthma remains limited. Most importantly, it is entirely unknown if asthma can impair vascular endothelial function, a key hallmark of cardiovascular disease risk. Thus, the goal of this study was to determine the effects of OVX on 1) asthma severity biomarkers and 2) aortic endothelial function in asthmatic mice. Given that asthma is worsened during the premenstrual phase when both estrogen and progesterone are low [12,13], we tested the hypothesis that lung cytokines and allergic biomarkers will be increased with OVX asthmatic mice, and that this will be associated with aortic vascular endothelial dysfunction.

## Materials and methods

### Ovalbumin sensitization and challenge

All animal care and experimental procedures were performed as outlined in the Guide for the Care and Use of Laboratory Animals (National Academy Press, 1996) and approved by the West Virginia School of Osteopathic Medicine (WVSOM) Institutional Animal Care and Use Committee (IACUC # 2022−1). Sham and OVX surgeries were conducted on 4-week-old female C57BL/6J mice (#000664) by the Jackson Laboratory (Bar Harbor, ME). This age was selected based on a previous study to avoid the mice from reaching sexual maturity [10]. Following surgery, animals were shipped to the WVSOM animal vivarium, where housing conditions consisted of a 12H:12H dark-light cycle, and temperature was maintained at 22° ± 2°. Animals were provided food (Inotive Teklad 2016: phytoestrogen-free diet), and water ad libitum. Once acclimatized, animals were randomly assigned to the following groups: 1) sham vehicle control (VC), 2) OVX VC, 3) sham asthma, and 4) OVX asthma.

**Fig 1:**
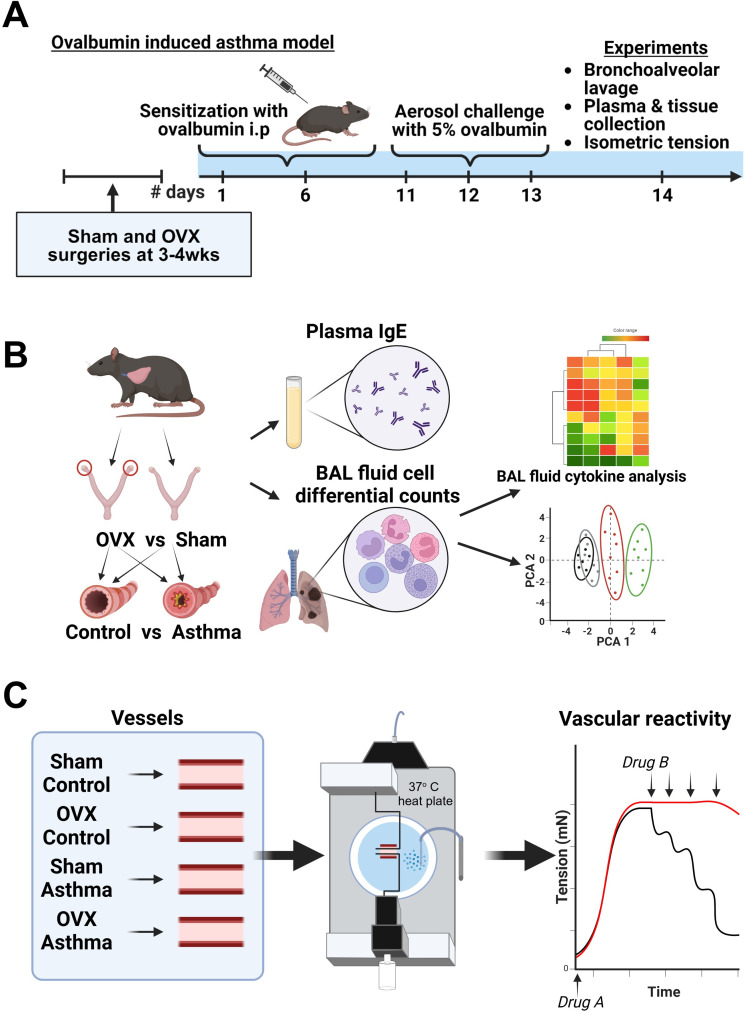
Schematic of experimental asthma protocol. **(A)** Mice were sensitized by intraperitoneal (i.p.) injection on days 1 and 6 with ovalbumin suspended in Imject alum which is used to stimulate an immune response. To keep an appropriate control, non-sensitized control mice only received the same volume of Imject alum without ovalbumin (Vehicle). On days 11-13, the allergen sensitized mice were exposed to challenges of aerosolized 5% ovalbumin in 0.9% saline while the vehicle control mice were exposed to 0.9% saline only for 20 minutes, twice a day, with a minimum interval of at least 6 hours using an ultrasonic nebulizer. **(B)** Schematic descriptions of experimental design and biochemical data collection are illustrated. **(C)** Schematic descriptions of experimental design and vascular physiology experiments are illustrated. (Created with BioRender.com).

Following acclimation, mice (age 6–8 wks) underwent induction of experimental asthma by ovalbumin sensitization, followed by ovalbumin aerosol challenges as described previously [14–16]. Briefly, mice were sensitized by i.p. injection on days 1 and 6 with ovalbumin (30 μg/mouse; Sigma-Aldrich) suspended in Imject alum (#77161, Thermo Fisher Scientific). Non-sensitized control animals only received the same volume of Imject alum (vehicle). On days 11–13, the allergen-sensitized mice were exposed to challenges of aerosolized 5% ovalbumin in 0.9% saline while the controls were exposed to 0.9% saline without ovalbumin for 20 minutes. The aerosol challenges were performed twice a day, once in the morning and once in the afternoon, using an ultrasonic nebulizer (De Vilbiss Healthcare), with a minimum interval of at least 6 hours. All groups followed the same schedule. (Fig 1). On the day of euthanasia, animals were given an intraperitoneal injection of sodium pentobarbital (50 mg/kg) to induce surgical anesthesia. After confirming the absence of response to painful stimuli, blood samples were collected into EDTA coated plasma tubes via cardiac puncture and centrifuged at 2000 x g for 10 min to isolate plasma. As a secondary confirmation of death, the heart and aorta were removed in accordance with our IACUC-approved protocol. Plasma samples were aliquoted and stored at −80 °C until experiments were performed. These approaches were selected based on extensive prior work from our laboratory demonstrating their sensitivity to ovalbumin-related impairments in tracheal relaxation, airway hyperresponsiveness, and elevated asthma-related biomarkers such as ovalbumin-specific IgE and IgG [14,15].

### Bronchoalveolar differential cell counts and ELISA

Bronchoalveolar (BAL) fluid samples were collected as described previously with a slight modification [15]. After the cardiac puncture, BAL fluid samples were collected by cannulating the trachea through a small incision with three lung lavages with 0.9% sterile saline (1 mL saline/lavage). BAL fluid volume was measured, and the samples were centrifuged for 7 min at 400 x g at 4 °C. The supernatant was aliquoted and saved in a −80 °C freezer for ELISA analyses. The pellet was resuspended in 200 uL of ACK lysis buffer (# A10492-01, Thermo Fisher Scientific) and incubated for 10 min on ice to remove the erythrocytes. After the red blood cell lysis, the sample was centrifuged for 7 min at 400 x g at 4 °C to collect the cell pellet. The cell pellet was then suspended in 0.5 mL 0.9% sterile saline. The total number of cells in the BAL fluid was counted by the TC20 automated cell counter (Bio-Rad Laboratories). For the differential cell analysis, the cell suspension was further diluted by the addition of 0.5 mL cold sterile saline and aliquoted into each well of the cytofunnel. Slides were then placed in the Cytospin 3 (Shandon Scientific) and spun at room temperature for 5 min at 800 rpm. After the cytospin and air drying for 24–36 h, cells were stained with Kwik-Diff staining solution (# 9990700, Shandon Scientific) based on the manufacturer’s protocol. Microscope images were captured using a Leica Aperio Versa 8. The different types of cells were counted under 40X magnification. A total of 300 cells were counted on each slide by two lab technicians who are not involved in the experiments.

For the cytokine measurements, BAL fluid samples were sent to Ampersand Biosciences, and 42 inflammatory markers were measured using Rodent MAP 4.0 (Ampersand Biosciences). Values falling below the lower limit of quantification were treated as zero values for statistical analysis. The remaining BAL fluid and plasma samples were analyzed by ELISA for anti-ovalbumin IgG1 (#500830, Cayman Chemical), and anti-ovalbumin IgE (#439807, Biolegend) according to the manufacturer’s instructions.

### Myograph experiments

Sodium Chloride (#S271) and Glucose (#D16) were obtained from Fisher Scientific. All other chemicals were purchased from Sigma-Aldrich Co. Endothelial-dependent relaxation was assessed in isolated aorta as previously described [17,18]. Aorta was dissected free of surrounding tissues and placed into the bath of the myograph chamber (DMT 620M, DMT) containing bicarbonate buffered physiological saline solution (PSS) containing (mM) 130 NaCl, 4.7 KCl (#P3911), 1.18 KH_2_PO_4_ (#P9791), 1.17 MgSO_4_ (#230391), 14.9 NaHCO_3_ (#S6297), 5.5 Glucose, 0.026 EDTA (#ED2SS), and 1.6 CaCl_2_ (#5080). Aortic rings were mounted on two stainless pins in organ baths that were oxygenated with 95% O_2_ plus 5% CO_2_ to maintain a pH of 7.4 with a temperature maintained at 37°C. Aortic rings were stretched in a stepwise manner to reach an optimal tension for 1 hour prior to functional studies. After equilibration, vessel viability was determined by potassium PSS containing 60 mM KCl (equimolar replacement of NaCl with KCl). After preconstruction to phenylephrine (#P6126 PE: 10^−6^ M), endothelial function was determined by measuring the relaxation of vessels in response to the cumulative addition of acetylcholine (#A6625 Ach: 10^−9^ to 10^−5^ M). To assess nitric oxide (NO), and the cyclooxygenase-mediated relaxation, ACh dose responses were repeated in the presence of the NOS inhibitor, NG-nitro-L-arginine methyl ester (#N5751 L-NAME: 10^−4^ M) or indomethacin (#I7378 Indo:10^-5^ M). Endothelium-independent relaxation was assessed by sodium nitroprusside (#71778 SNP) dose-response (10^−10^ to 10^−6^ M) after preconstruction with PE (10^−6^ M). Isometric tension was continuously recorded using a force displacement transducer connected with a Transbridge Transducer Amplifier (Power Lab 8/35, AD Instruments). The data were digitized at 100 Hz and stored using Lab Chart (LabChart, AD Instruments).

### Statistical analysis

Data are presented as the mean ± standard error of the mean (SEM) using GraphPad Prism (GraphPad Software, San Diego, CA, USA). BAL differential count data were analyzed by a two-way ANOVA (factor 1, sham vs OVX; factor 2 different cell types) followed by Sidak’s multiple comparisons test. For the cytokine analysis, unsupervised hierarchical cluster analysis was conducted by ClustVis (http://biit.cs.ut.ee/clustvis/) on cytokine expression values from BAL fluid for data visualization [19]. The principal component analysis was performed using GraphPad Prism. Cytokine data were analyzed by a two-way ANOVA (factor 1, sham vs. OVX; factor 2 VC vs. Asthma). To control the false discovery rate in the multiple hypothesis testing, the Benjamini-Hochberg procedure with a false discovery rate of 5% was used to adjust raw *p*-values obtained from two-way ANOVA [20].

Myograph dose-response curves were created as recommended by the guidelines [21]. Area under the curve (AUC) and E_max_ (maximum response) were analyzed by one-way ANOVA followed by Bonferroni post hoc tests to determine differences between the groups. Other parametric statistical comparisons were made using one- or two-way ANOVA with Bonferroni post hoc tests as appropriate using GraphPad Prism (GraphPad Software, San Diego, CA, USA).

## Results

Animal characteristics of the groups are presented in Table 1. The mean uterine weight in OVX mice was approximately 90% lower than in ovary intact mice, confirming depletion of sex hormones (sham VC 76.4 ± 8.7, OVX VC 5.6 ± 0.5, sham asthma 54.8 ± 10.9, OVX asthma 6.3 ± 0.3 mg, *p* < 0.05). Body mass, thymus, and sleep weights were measured as indicators of general health, surgical stress, and systemic immune alterations. Neither ovalbumin-induced asthma nor OVX surgery had any effect on body mass, thymus, or spleen weight.

**Table 1:** Animal characteristics.

Groups	Sham VC	OVX VC	Sham Asthma	OVX Asthma
**Age (wks)**	9.3 ± 0.3	8.9 ± 0.3	9.4 ± 0.3	9.2 ± 0.3
**Body weight (g)**	19.4 ± 0.46	19.8 ± 0.41	20.3 ± 0.61	19.9 ± 0.32
**Uterus weight**	76.4 ± 8.7	5.6 ± 0.5*	54.8 ± 10.9	6.3 ± 0.3**
**Spleen weight (mg)**	165.6 ± 24.8	177.3 ± 14.5	150.8 ± 11.2	202.3 ± 19.2
**Thymus weight (mg)**	66.3 ± 4.86	57.7 ± 6.55	69.3 ± 5.16	65.9 ± 5.91
**Kidney weight (mg)**	130.1 ± 6.16	137.5 ± 9.61	127.2 ± 5.28	128.1 ± 4.51
**BAL total cells (10**^**5**^)	7.4 ± 1.8	6.4 ± 1.5	34.6 ± 9.6*	31.1 ± 6.0**
**Monocytes (10**^**5**^)	6.7 ± 0.2	6.1 ± 1.5	8.3 ± 2.7	3.2 ± 1.0**
**Eosinophils (10**^**4**^)	0.1 ± 0.1	0.8 ± 0.3	199.8 ± 47.4*	238.6 ± 43.3**
**Lymphocytes (10**^**4**^)	5.6 ± 2.0	1.0 ± 0.3	12.4 ± 3.5	20.4 ± 10.0
**Neutrophils (10**^**4**^)	0.5 ± 0.2	0.5 ± 0.2	44.8 ± 11.5*	21.1 ± 5.2

Values are mean ± standard error of the mean. Sham vehicle control (VC) and ovariectomized (OVX) VC mice were sensitized with Imject alum followed by saline aerosol challenges. While Sham Asthma and OVX Asthma mice were sensitized by i.p. injection with ovalbumin suspended in Imject alum followed by challenges of aerosolized 5% ovalbumin in 0.9% saline. * *p* < 0.05 vs Sham VC; ** *p* < 0.05 vs OVX VC (n = 8 from each group).

### Lung allergic responses are altered with OVX in C57BL/6J mice

**Fig 2:**
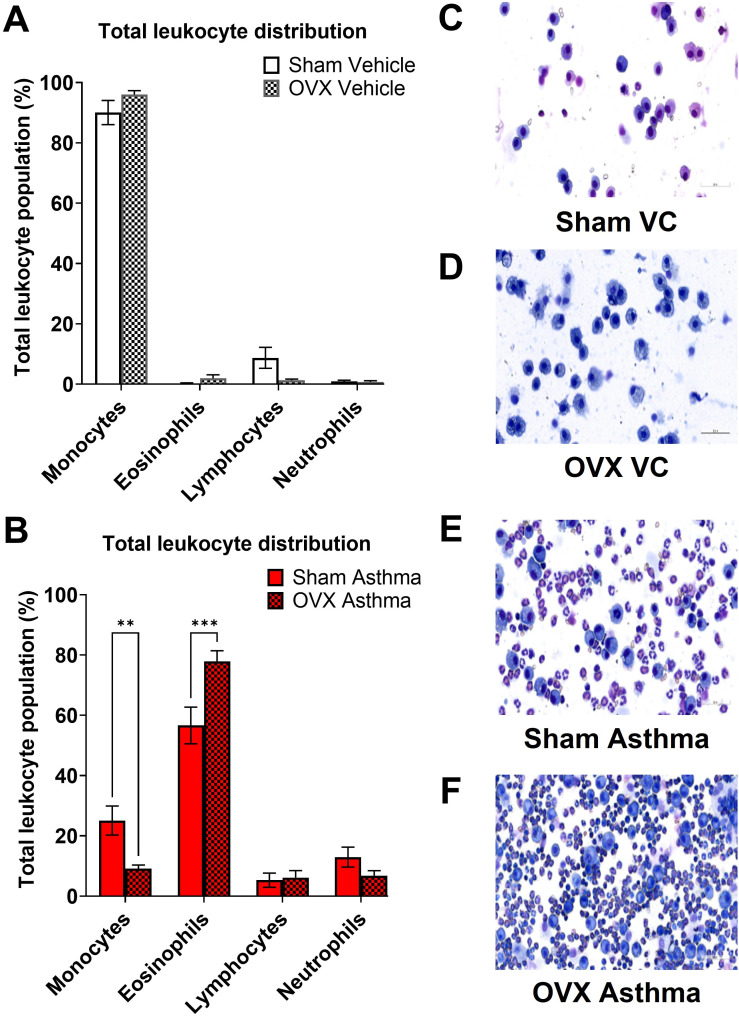
Assessment of differential leukocyte counts in BAL fluid from sham and ovariectomized (OVX) asthmatic mice. Differential counts for monocytes, eosinophils, lymphocytes, and neutrophils were performed on Kwik-diff–stained cells. **(A)** Group data showed that OVX itself did not change the total leukocyte populations in BAL fluid. **(B)** Asthma significantly changed total leukocyte distribution in BAL fluid and OVX significantly augmented the eosinophil population. Representative images for (C) sham female vehicle control (VC) and (D) for OVX VC are shown. Representative images (E) sham female asthma and **(F)** OVX asthma showed extensive eosinophilia. ** and *** indicated p < 0.01 and p < 0.001 by two-way ANOVA, respectively (n = 8 mice per group).

To investigate the effects of OVX on the levels of asthma biomarkers, BAL fluid samples were analyzed. After 2 weeks of ovalbumin sensitization and challenge, total cell counts of the sham asthma and OVX asthma groups were increased by 368 and 386% compared to the relative control groups, respectively (sham VC 7.4 ± 1.8, OVX VC 6.4 ± 1.5, sham asthma 34.6 ± 9.6, OVX asthma 31.1 ± 6.0 10^5^ cells, *p* < 0.05, Table 1). Fig 2 shows representative images of BAL Diff-Quik stain from each group. OVX itself had no effect on differential counts of BAL cells in vehicle treatment (Monocytes, Eosinophils, Lymphocytes, and Neutrophils: sham VC 91 ± 2.8, 0.2 ± 0.1, 8.1 ± 2.5, 0.7 ± 0.3 vs. OVX VC 96 ± 0.8, 1.4 ± 0.7, 1.7 ± 0.4, 0.8 ± 0.3%, Fig 2A). BAL fluid eosinophil percentage was significantly higher in OVX asthma mice (sham asthma: 57 ± 6.1 vs. OVX asthma: 78 ± 3.5%, Fig 2B, *p* < 0.05).

**Fig 3:**
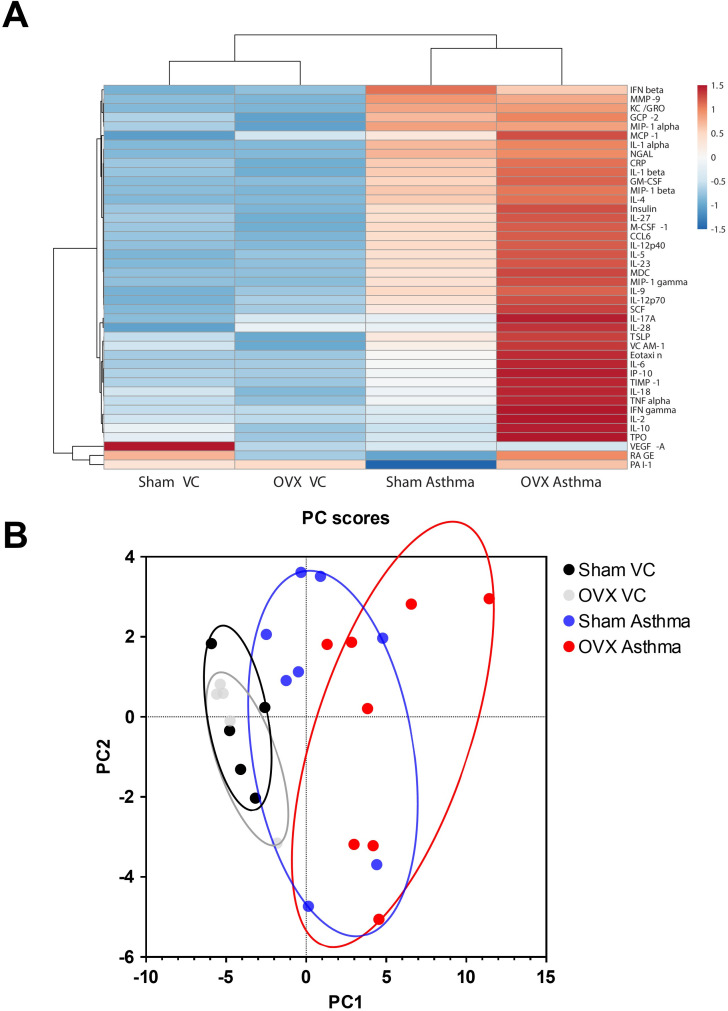
Inflammatory biomarkers in BAL fluid after 2 weeks of ovalbumin-induced asthma in C57BL/6 mice. **(A)** Hierarchical clustering analysis showed the differentially expressed 42 cytokines for each group. (n = 5 for control groups and n = 8 for asthma groups). **(B)** The plot of the principal component (PC) scores from PC1 and PC2 were presented using PCA analysis of 42 cytokines from each animal (sham VC; black dots, OVX VC; gray dots, sham asthma; blue dots, and OVX asthma; red dots).

To further confirm if OVX asthmatic mice had an altered lung allergic response, we also measured cytokine levels in BAL fluid samples. Fig 3 shows a heatmap of 42 inflammatory markers and the results of the principal component analysis (PCA). Unsupervised hierarchical clustering analysis revealed distinct BAL fluid cytokine profiles between sham vs OVX asthma groups (Fig 3A). PCA with parallel analysis identified the 3 principal components (PC) that explain 74.3% of the total variance in BAL fluid 42 cytokine data (PC1 47.7, PC2 15.4, and PC3 11.2%, supplement data). The plot of the PC scores from PC1 and PC2 demonstrated that sham VC and OVX VC were distributed closely, while the differential patterns of responses to asthmatic insults in sham asthma and OVX asthma groups with significant differences in PCA1 scores (sham VC −4.11 ± 0.59, OVX VC −4.55 ± 0.69, sham asthma 0.71 ± 0.92, OVX asthma 4.71 ± 1.10, respectively. *p* < 0.05, supplemental data). Among the 42 cytokines, 32 cytokine expressions changed by asthma status (*p* < 0.05, supplemental data). The interaction effect of asthma and OVX identified 3 statistically significant markers: MIP-1 gamma (sham asthma 4.51 ± 0.61 vs. OVX asthma 7.50 ± 0.56 ng/mL, *p* < 0.05), TPO (sham asthma 0.0115 ± .002 vs. OVX asthma 0.032 ± .004 ng/mL, *p* < 0.05), and Eotaxin (sham asthma 46.1 ± 8.9 vs. OVX asthma 102.6 ± 13.6 pg/mL, *p* < 0.05).

**Fig 4:**
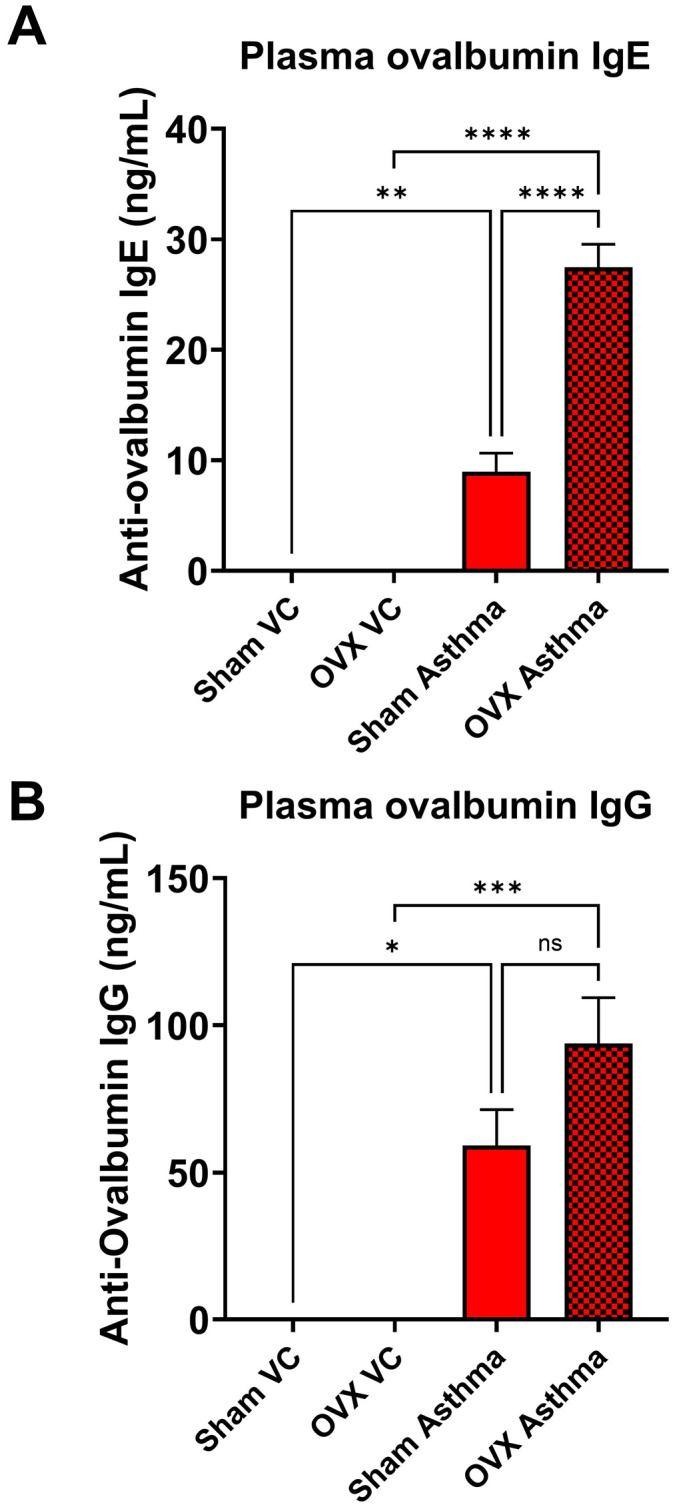
Asthma severity biomarkers in plasma after 2 weeks of ovalbumin-induced asthma in C57BL/6 mice. **Plasma ovalbumin-IgE and -IgG levels were quantified by ELISA from sham VC, OVX VC, sham asthma, and OVX asthma groups. (A)** Plasma ovalbumin-IgE levels were significantly higher in the OVX asthma group than in the sham asthma group. **(B)** A trend toward an increase in ovalbumin-IgG levels was seen in the OVX asthma group, but this increase was not statistically significant (p = 0.09).*, **, *** and **** indicated p < 0.05, < 0.01, < 0.001 and < 0.0001 by one-way ANOVA, respectively (n = 4-10 mice per group).

To determine the severity of asthma, the levels of allergen-specific IgE and -IgG1 in plasma and BAL fluids were measured. Both sham and OVX asthmatic mice showed a significant increase in plasma ovalbumin-IgE (Fig 4A, *p* < 0.05). Interestingly, ovalbumin-IgE levels of OVX asthma were 206% higher than the sham asthma group (sham asthma vs OVX Asthma, 8.98 ± 1.66, vs. 27.5 ± 2.07 ng/mL, *p* < 0.05). Although BAL fluid ovalbumin-IgE levels were higher in both asthmatic groups, this finding was not statistically significant. (S1 Fig.). Similarly, plasma ovalbumin-IgG1 levels of sham asthmatic mice were significantly increased when they were compared with sham control mice (sham VC vs sham asthma, 0.06 ± 0.04, vs. 59.1 ± 12.2 ng/mL, Fig 4B, *p* < 0.05). Plasma ovalbumin-IgG1 levels of the OVX asthma group were also significantly increased (OVX VC vs OVX Asthma, 0.46 ± 0.31, vs. 93.8 ± 15.6 Fig 4B, *p* < 0.05). Although statistically insignificant, ovalbumin-IgG1 levels of OVX asthma were 59% higher than sham asthmatic mice. (59.1 ± 12.2, vs. 93.8 ± 15.6, Fig 4B, *p* = 0.09). BAL fluid ovalbumin-IgG1 levels from both asthmatic groups were also elevated, however, there were no statistical differences between them (sham asthma vs OVX asthma: 53.0 ± 8.94 vs. 96.1 ± 20.6 ng/mL, S1 Fig, *p* = 0.08). Taken together, these biochemical data from BAL fluid, lung, and plasma demonstrate classic asthma-related pathological changes and suggest that OVX may promote a shift toward a TH2-skewed immune response rather than increased lung inflammation, as supported by altered BAL leukocyte populations, elevated IgE levels, and increased Th2 cytokine expression.

### Ovalbumin-induced asthma appears to impair endothelial-dependent relaxation in female mice, but not endothelial-independent relaxation

**Fig 5:**
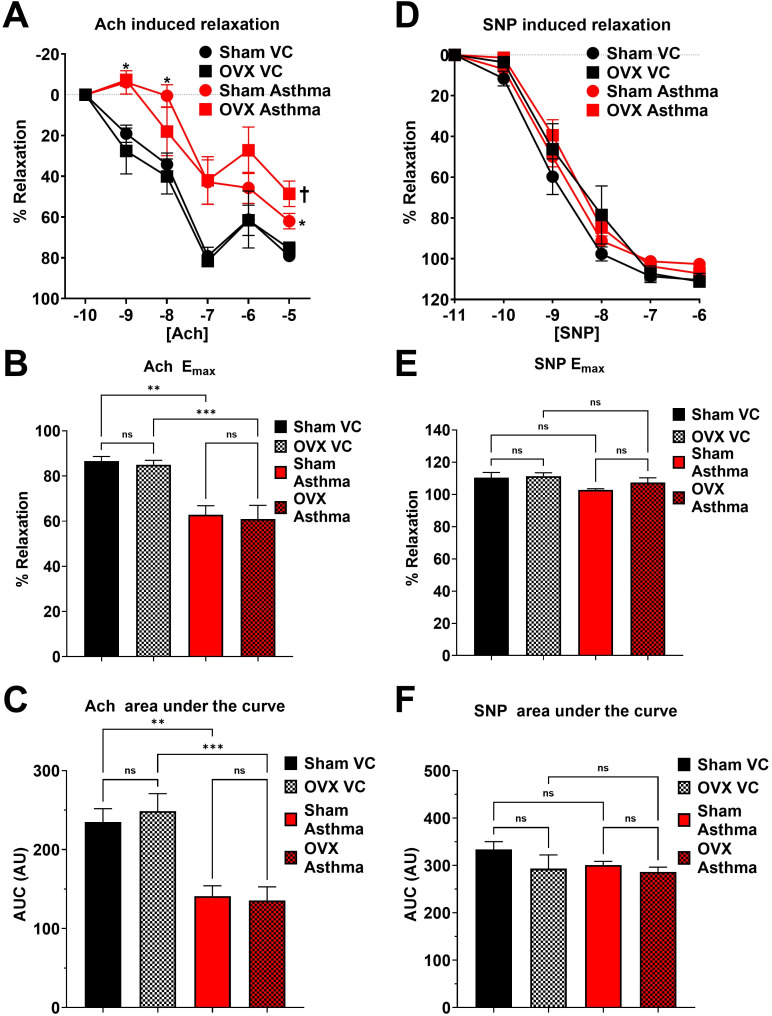
Endothelium-dependent and independent relaxation responses of aorta. Isometric tension experiments were performed on aortic rings from each group. (A & D) Aortic rings were preconstructed with phenylephrine (10 ^−6^ M) and then exposed to cumulative doses of acetylcholine (Ach) and sodium nitroprusside (SNP) for the assessment of endothelial-dependent and -independent relaxation. * and † indicated p < 0.05 compared to the relative control groups by two-way ANOVA, respectively (n = 8 mice per group). (B & C) Group data for maximal Ach-induced relaxation (Emax) and the area under the curve (ACU) showed that asthma significantly decreased Emax and ACU. (E & F) Group data for SNP Emax and ACU indicated no difference among the groups. ** and *** indicate p < 0.01, <and 0.001 by one-way ANOVA (n = 8 mice per group).

The lungs receive the entire cardiac output, and the aorta is the first vascular system exposed to any lung inflammation that may then spread to the systemic circulation. Thus, we selected the aorta as the vessel of choice for isometric tension experiments. Isometric experiments were used to determine the effect of OVX and asthma on aortic rings. Maximal acetylcholine (Ach) induced aortic relaxation was reduced by 23.8 ~ 24.1% in both sham female and OVX asthmatic mice compared with respective vehicle control mice (sham VC vs. sham Asthma: 86.6 ± 2.03 vs. 62.8 ± 4.03%, *p* < 0.05, and OVX VC vs. OVX asthma: 85.0 ± 1.93 vs. 60.9 ± 6.11%, *p* < 0.05, n = 8 each group, Fig 5B). Similarly, AUC was lower in both asthmatic aorta than in the control groups (sham VC vs sham asthma: 234 ± 16.9 vs. 141 ± 13.0, *p* < 0.05, and OVX VC vs. OVX asthma: 249 ± 17.3 vs. 136 ± 17.3 AU, *p* < 0.05, Fig 5C). However, there were no statistically significant differences between sham asthma and OVX asthma (141 ± 13.0 vs 136 ± 17.3 AU, p = 0.99, Fig 5C). On the other hand, smooth muscle sensitivity to nitric oxide (NO) assessed by the response to the nitric oxide donor (sodium nitroprusside) was not affected in all groups (Fig 5D, E, and F, n = 8). These results demonstrate that aortic endothelial function, at least in response to pharmacological stimulation by Ach, is reduced in both female asthmatic mice groups without changing vascular smooth muscle sensitivity to NO. Interestingly, there appeared to be no difference in the severity of aortic endothelial dysfunction between sham asthma and OVX asthma mice.

**Fig 6:**
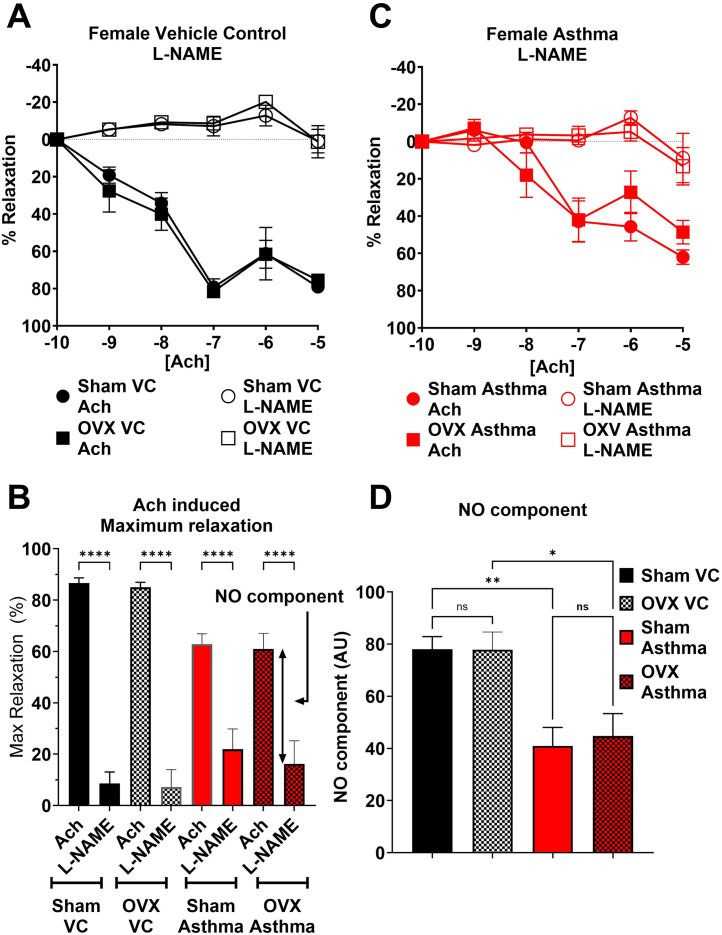
Contribution of NO in Ach-induced relaxation in asthmatic mice. Isometric tension experiments were performed on aortic rings in the presence or absence of L-NAME (10^−4^ M). Aortic rings from control groups (A) and asthma groups (C) were pre-contracted with phenylephrine (10^−6^ M), and relaxation responses were assessed with increasing concentrations of Ach (10^−9^ to 10^−5^ M). (B) Group data showed that Ach induced relaxation was significantly inhibited by L-NAME. (D) Nitric oxide (NO) components were significantly decreased in both asthmatic groups, but there were no differences between sham asthma and OVX asthma. *, ** and **** indicated *p* < 0.05, < 0.01, and < 0.0001 by one-way ANOVA (n = 8 mice per group).

**Fig 7:**
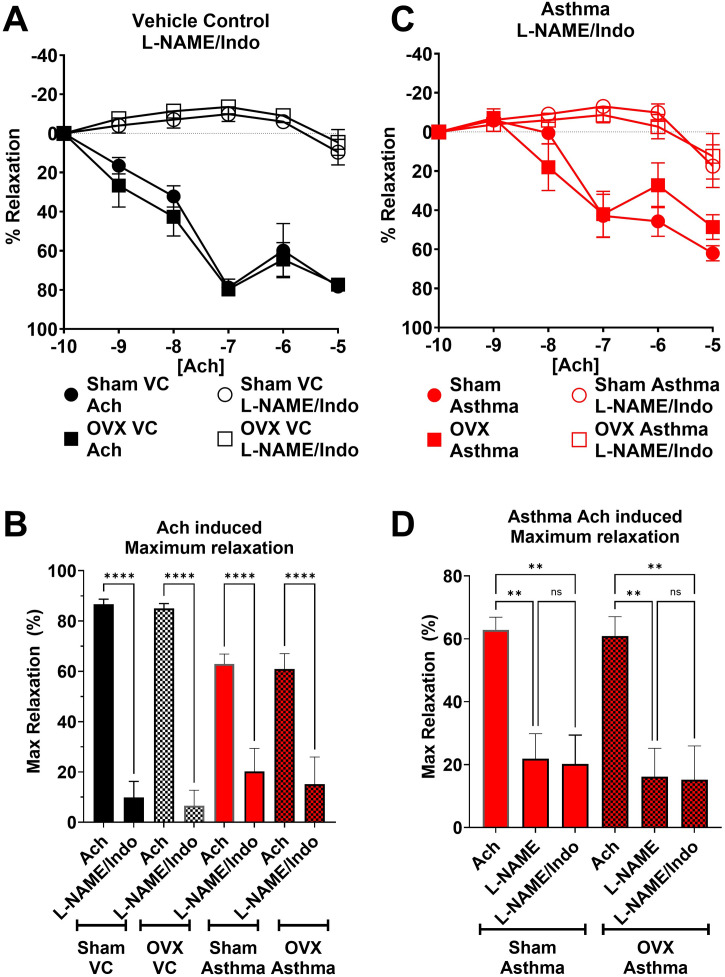
Contribution of prostacyclin (PGI_2_) in Ach-induced relaxation in asthmatic mice. Isometric tension experiments were performed on aortic rings in the presence or absence of L-NAME (10^−4^ M) and Indomethacin (Indo: 10^−5^ **M)**. Aortic rings from control groups (A) and asthma groups (C) were pre-contracted with phenylephrine (10^−6^ **M)**, and relaxation responses were assessed with increasing concentrations of Ach (10^−9^ to 10^−5^ **M)**. **(B)** Group data showed that Ach-induced relaxation was significantly inhibited by L-NAME and Indo. **(D)** Ach-induced maximum relaxation was not different between L-NAME only vs. L-NAME plus Indo in both asthma groups. ** and **** indicated *p* < 0.05, and < 0.0001 by one-way ANOVA (n = 8 mice per group).

Figs 6 and 7 show the relative contributions of NO and prostacyclin to the endothelial-dependent relaxation of aorta from both groups of asthmatic mice. In these paired experiments, Ach dose responses were performed before and after 20 minutes of incubation with L-NAME (10^-4^M). In the aortic rings from both sham and OVX VC groups, endothelial-dependent relaxation to Ach was almost abolished by L-NAME (Fig 6A and B). In the aorta of both sham and OVX asthmatic mice, L-NAME markedly inhibited the endothelial-dependent relaxation in response to Ach (Fig 6B and C). NO components were calculated by the differences in relaxation in each aortic ring before and after L-NAME. Compared to each control group, NO components were 48% and 42% less in sham asthma and OVX asthma groups (sham VC, OVX VC, sham asthma, and OVX asthma: (78.0 ± 4.9, 77.9 ± 6.8, 40.9 ± 7.1, 44.8 ± 8.6, AU, n = 8, *p* < 0.05, Fig 6D). However, there were no differences in NO components between sham and OVX asthmatic mice. A similar reduction of Ach induced relaxation was observed in the presence of L-NAME and indomethacin (10^-5^M) indicating that the contribution of prostacyclin is little or negligible (Fig 7). These data suggest that NO plays a large role in the relaxation to Ach in asthmatic aorta and the NO contribution to relaxation in the aorta is reduced in ovalbumin-induced asthmatic female mice. However, there seems to be no statistical difference between sham and OVX asthma groups (Fig 6D). Taken together, the asthma-associated impairment in endothelial dysfunction resulted from a reduction in NO bioavailability, evidenced by a smaller reduction in maximum relaxation in response to Ach in asthmatic aorta incubated with a NO synthase inhibitor.

## Discussion

The purpose of this study was to determine 1) if OVX worsens lung allergic responses in ovalbumin-induced asthmatic mice compared to ovary-intact asthmatic mice, and 2) if aortic endothelial dysfunction, a key marker of cardiovascular disease (CVD), exists in female asthmatic mice. Asthma symptoms are known to be impacted by female sex hormonal fluctuations and tend to worsen during phases of the ovarian cycle when circulating estrogen and progesterone levels are the lowest [3]. Thus, we used the OVX model as a surrogate of ovarian hormone deficiency to mimic this low hormone state without cycling. Our data demonstrated that OVX in ovalbumin-induced asthmatic C57BL/6 mice increased the percentage of eosinophils in BAL fluid, cytokines, and plasma ovalbumin-IgE compared to ovary-intact asthmatic mice. Although both OVX and ovary-intact asthmatic mice exhibited aortic endothelial dysfunction in the aorta, there were no differences in endothelial-dependent relaxation between the groups. Some of these data are in agreement with previous studies showing that in asthmatic mice, the removal of the ovaries causes the development of more severe lung inflammation and eosinophilia [10,11]. In our study, these lungs’ eosinophilia and increased inflammation due to experimental asthmatic insults were parallel with impaired vascular endothelial dysfunction in the aorta. It is important to note that this study cannot provide a mechanistic understanding of how lung allergic responses cause aortic endothelial dysfunction. Interestingly, OVX asthmatic mice did not show more severe aortic endothelial dysfunction; however, to our knowledge, these findings have not been reported before.

### OVX lung inflammation

Although it seems clear that female sex hormones play a role in asthma severity, contradictory findings in the literature highlight the complexity of preclinical asthma studies. A previous study by Draijer et. al., demonstrated that OVX asthmatic mice exhibited significantly higher eosinophils, macrophages, B lymphocytes, and IgE in BAL fluid compared with sham asthmatic mice [10]. On the other hand, Antunes et. al., reported that the total number of cells, eosinophils, neutrophils, mononuclear cells, IL-4, IL-5, and TGF-beta levels in BALF was lower in OVX asthmatic mice compared with sham female asthmatic mice [9]. Furthermore, Riffo-Vasquez et al. demonstrated that the effects of OVX varied depending on the timing of OVX surgeries (prior sensitization vs post sensitization) [11]. In our study, BAL cell differential count data from OVX asthmatic mice showed a significantly increased percentage of eosinophils compared with sham asthmatic mice. These data were associated with increased general cytokine levels in BAL fluid and plasma ovalbumin IgE, a circulating biomarker of asthma severity. These data are consistent with previous studies [10,11]. We do not have a clear explanation of the contradictory findings to the study [9],but methodological differences such as different strains of mice, and adjuvant vs. adjuvant-free sensitizations, could account for the differences between the studies. Indeed, BALB/c and C57BL/6 mice show distinctly different physiological, immunological, and molecular responses in ovalbumin-induced asthma models [22,23]. BALB/c mice show increased airway hyperresponsiveness, driven by elevated Th2 cytokines such as IL-4, IL-5, and IL-13 in lung tissue, along with increased mast cell infiltration. In contrast, C57BL/6 mice display relatively weaker airway hyperreactivity, but develop stronger airway inflammation characterized by higher numbers of BAL eosinophils and neutrophils with higher levels of chemokines such as CCL11 and CCL5. Because BALB/c and C57BL/6 exhibit different inflammatory responses and allergen sensitivity, direct comparisons across different strains are inherently challenging. Overall, the complexity of female sex hormone-related immune responses in allergic mice requires further studies.

### Asthma, cardiovascular diseases (CVD), and the female population

There is emerging evidence suggesting that female asthmatic patients may have an increased risk of CVD [4,5]. Female sex hormones seem to be related not only to asthma severity [24], but also to vascular function [25]. Previous studies have demonstrated that experimental asthmatic insults (ragweed sensitization and challenge) impaired aortic endothelial function in male Balb/c mice [26,27]; however, these studies used only male animals. This is an important issue in current preclinical studies because sex is an important biological variable in the preclinical cardiovascular research field [28]. We found that maximal acetylcholine (ACh) -induced aortic relaxation was reduced by 24% in both sham and OVX asthmatic mice groups compared with respective control mice after ovalbumin sensitizations and challenges (Fig 5A and B, *p* < 0.05). These effects were endothelial-dependent because nitric oxide (NO) donor sodium nitroprusside responses to relaxation were not different among groups (Fig 5D and E). We did notice that both vehicle control groups had lower Ach-induced relaxation compared to native mice (non-sensitized mice, S2 Fig.). Based on these observations, we believe Imject alum has a mildly toxic effect on endothelial cells.

Further pharmacological dissection of asthma-associated impaired aortic relaxation suggests a reduction in NO bioavailability, evidenced by a smaller contribution of NO to Ach-induced relaxation in the aorta (Fig 6B and D, *p* < 0.05). The combined inhibition of NO and prostaglandins by L-NAME and indomethacin did not produce a greater reduction in Ach-induced relaxation compared with L-NAME alone (Fig 7D). This suggests that prostaglandins do not significantly contribute to the impaired Ach-mediated vasorelaxation and supports the notion that reduced NO-mediated aortic relaxation is the primary mechanism underlying the aortic endothelial dysfunction observed in asthma.

Although there appears to be a trend toward greater endothelial dysfunction in OVX asthmatic mice based on acetylcholine dose-response data (Fig 5), no significant differences were observed between the sham asthma and OVX asthma groups. These unexpected results may be related to the timing of the vascular reactivity experiments. A recent report suggested that endothelial dysfunction is highly dependent on spatial and temporal heterogeneities [29]. In our experiment, pulmonary inflammation was assessed 24 hours after the final challenge; however, this time point may not have been optimal for detecting functional alterations in aortic endothelium. We selected this interval based on prior work in which airway responsiveness was shown to be altered at 24 hours using unrestrained whole-body plethysmography [14,15]. Our data suggest that inflammatory processes initiated in the lungs can lead to aortic endothelial dysfunction. Although we were unable to directly establish the temporal sequence between circulating biomarkers and endothelial physiological alterations, it is plausible that biomarker changes precede measurable dysfunction in the aortic endothelium.

Since we observed that OVX increases lung inflammation, but the reduction in NO‑mediated aortic relaxation is similar between Sham‑Asthma and OVX‑Asthma groups, NO may not be the only contributor to impaired asthma‑related ACh‑induced relaxation. We have previously shown the presence of large‑conductance Ca² ⁺ /voltage‑sensitive K⁺ channels, Kv_1.5_, and K_ATP_ currents in the mouse aorta [17]. Because endothelial derived hyperbolizing factor (EDHF) often acts by activating these K⁺ channels in vascular smooth muscle cells to relax the vessels, it is possible that the contribution of K⁺ channel mediated vasodilation may be altered in the OVX‑asthma condition. Additionally, endothelin‑1 (ET‑1) may also contribute to the decreased ACh‑induced relaxation observed in the asthmatic mouse aorta. ET‑1 signaling is known to be upregulated in asthma and contributes to bronchoconstriction [30]. In addition to its effects in the airways, ET-1 also induces endothelial activation by increasing the expression of adhesion molecules such as VCAM-1 and ICAM-1, enhancing leukocyte adhesion and vascular inflammation. Although the role of ET-1 in asthma‑associated vascular dysfunction is not well studied, ET‑1 is known to impair endothelium‑dependent vasodilation, as demonstrated by reduced flow‑mediated dilation [31]. Thus, ET‑1 signaling may be upregulated in asthma, leading to increased endothelial activation and reduced ACh‑induced relaxation. Further studies will be needed to determine the extent to which EDHF or ET‑1 signaling contributes to vascular dysfunction in the asthmatic vasculature.

Overall, our data indicate that the decrease in NO-mediated aortic relaxation does not differ substantially between sham asthma and OVX asthma. Nonetheless, to our knowledge, no previous studies have described the relationship between ovalbumin-induced pulmonary inflammation and vascular endothelial dysfunction in OVX mice. Finally, it is important to note that endothelial dysfunction observed in large conduit arteries should not be generalized to peripheral microvascular outcomes or overall vascular dysfunction. Thus, the pathophysiological significance of the apparent differences in lung allergic responses and aortic endothelial dysfunction in OVX asthmatic mice awaits further clarification. Future studies will be necessary to investigate the temporal dynamics of vascular endothelial dysfunction to more precisely define the relationship between pulmonary and vascular inflammation in asthmatic mice.

### Limitations

In this study, we were not able to provide pulmonary function test (PFT) data for each animal. Mice undergoing the PFT would be exposed to methacholine (Mch), which is significantly more stable than acetylcholine due to its resistance to acetylcholinesterase. Vascular reactivity and PFT cannot be assessed in the same mice. Additional sets of animals, specifically for the PFT, would be required. To avoid these issues, we chose plasma IgE, BAL fluid eosinophil, and inflammatory cytokines as asthma severity markers because these markers are used clinically [32,33]. Also, the acute allergen challenge used in this study may limit the detection of cumulative vascular dysfunction, which in clinical asthma likely develops with prolonged inflammatory exposure. Thus, the short-term protocol we used may partly explain the modest vascular effects observed. Future studies using chronic, clinically relevant allergen models (e.g., house dust mite) may better capture long-term vascular consequences. Another limitation of our study was the lack of measurements for circulating estrogen or progesterone due to technical restrictions. However, the depletion of female sex hormones was evident by significantly reduced uterine weight (Table 1), consistent with a prior report [34]. Finally, our study would be strengthened with follow-up experiments with 1) estrogen only, 2) progesterone only, and 3) estrogen and progesterone replacement groups to potentially reverse the effects we observed from OVX asthma groups.

## Conclusion

Our data demonstrated that OVX asthmatic mice developed exacerbated allergic lung responses. This worsening of lung inflammation may suggest a potentially protective role of ovarian hormones on the lungs. While aortic endothelial dysfunction was observed in both asthmatic mice groups, OVX did not worsen vascular function in asthmatic mice as anticipated. Thus, further studies are necessary to determine whether the timing of assessment influences vascular dysfunction and to identify which factors regulating vascular function and/or other vascular beds are associated with the lung inflammation we observed.

## Supporting information

S1 Fig The effects of OVX and asthma on BAL fluid ovalbumin specific-Ig E and-IgE. (TIFF)

S2 Fig The effects of adjuvant Imject alum on endothelial-dependent relaxation. (TIFF)

## Abbreviations

CVD: Cardiovascular diseases
OVX: Ovariectomy
VC: Vehicle control
BAL fluid: Bronchoalveolar lavage
PC: Principal components
Ach: Acetylcholine
PE: Phenylephrine
L-NAME: NG-nitro-L-arginine methyl ester
SNP: Sodium nitroprusside
NO: Nitric oxide
AUC: Area under the curve
Emax: Maximum response.
