# Supplementary figures and images for: Characterization of aortic endothelial dysfunction in ovariectomized allergic asthmatic mice
Source: PLoS One. 2026 Jul 22;21(7):e0352768. doi: 10.1371/journal.pone.0352768 (PMC13390832; doi:10.1371/journal.pone.0352768)

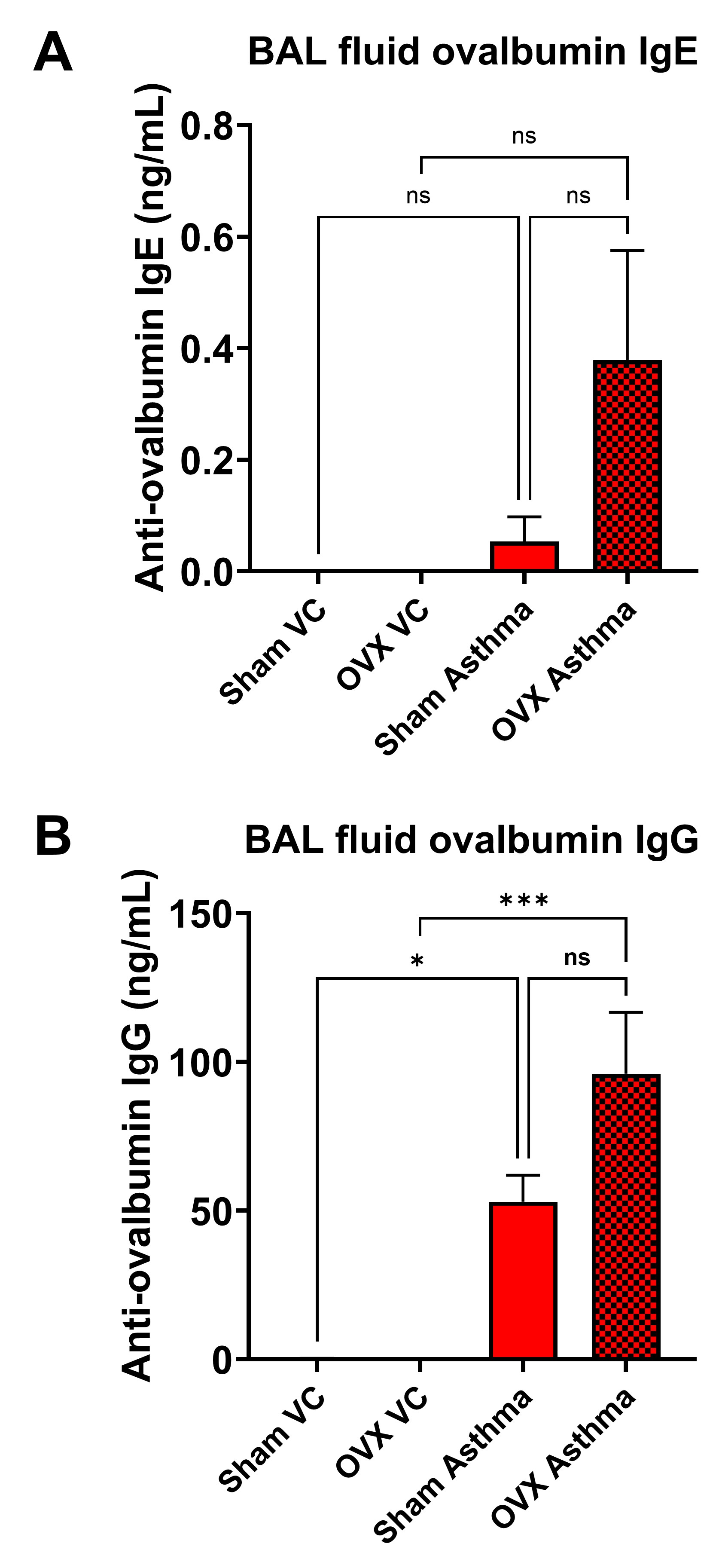

Supplement: S1 Fig — (TIFF) [file pone.0352768.s001.tiff]

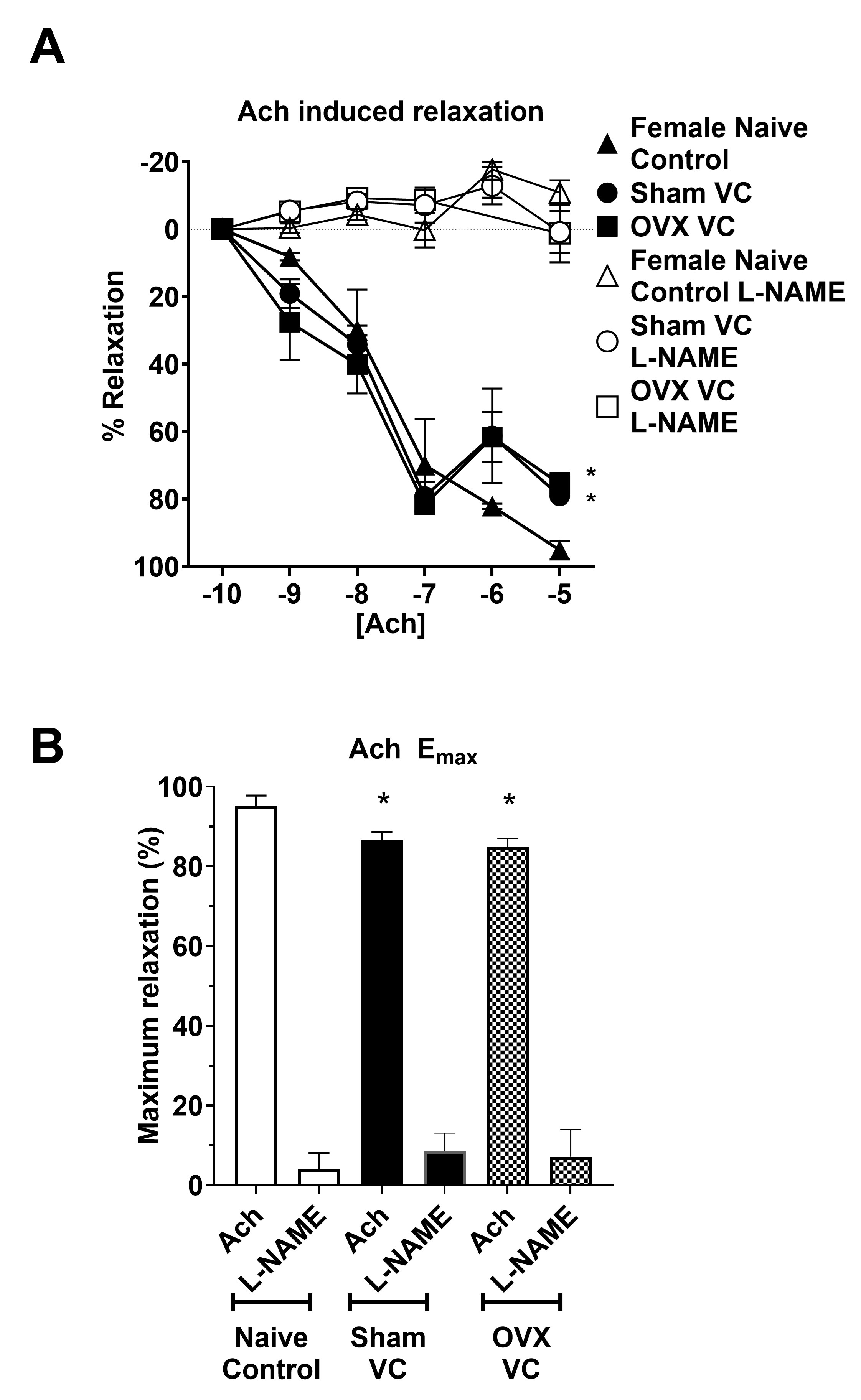

Supplement: S2 Fig — (TIFF) [file pone.0352768.s002.tiff]
